# STEMI patients receiving percutaneous coronary intervention for a culprit lesion with coronary artery bifurcation—efficacy and safety of the jailed semi-inflated balloon

**DOI:** 10.3389/fcvm.2023.1132062

**Published:** 2023-06-30

**Authors:** Tzu-Hsiang Lin, Kuan-Ju Chen, Yu-Cheng Hu, Keng-Hao Chang, Chih-Hung Lai, Tsun-Jui Liu, Wen-Lieng Lee, Chieh-Shou Su

**Affiliations:** ^1^ Cardiovascular Center, Taichung Veterans General Hospital, Taichung, Taiwan; ^2^ Department of Emergency Medicine, Taichung Veterans General Hospital, Taichung, Taiwan; ^3^ Department of Internal Medicine, Cheng Ching Hospital, Taichung, Taiwan; ^4^ Institute of Clinical Medicine, Department of Medicine, National Yang-Ming University School of Medicine, Taipei, Taiwan; ^5^ Department of Medicine, National Yang Ming University School of Medicine, Taipei, Taiwan

**Keywords:** ST-elevation myocardial infarction, primary percutaneous coronary intervention, side branch protection, jailed semi-inflated balloon technique, coronary artery bifurcation

## Abstract

**Background:**

We aimed to evaluate the efficacy and safety of the ‘jailed semi-inflated balloon technique’ (JSIBT) for side branch (SB) protection in STEMI patients with a culprit lesion involving a coronary artery bifurcation while undergoing emergent percutaneous coronary intervention (PCI).

**Methods:**

We treated between Jan, 2011 and Jun, 2020, a total of 264 STEMI patients with a culprit lesion that involved a coronary artery bifurcation using primary PCI. In 30 patients, SB was protected by JSIBT (the JSIBT group). In 234 patients, SB was either protected or not protected by a placed wire (the non-JSIBT group).

**Results:**

In both groups, after PCI procedure, TIMI flows of main vessel (MV) and SB were increased significantly compared with their measurements before the procedure. TIMI flows of post-procedural MV were similar between the two groups. In the JSIBT group, TIMI flows of SB both peri-procedure and post-procedure measurements were significantly greater than the non-JSIBT group. Despite a higher incidence of SB dissection in the JSIBT group, no inter-group difference was found in their total SB complications (like SB dissection, SB occlusion, wire entrapment or balloon rupture/entrapment). While JSIBT was an independent predictor for the SB TIMI 3 flow measured at the end of primary PCI, it was not an independent predictor for SB complications.

**Conclusion:**

The use of JSIBT as a method of SB protection during primary PCI not only provided better SB protection, but it also had a similar rate of SB complications compared with those with or without prior application of SB wire. This technique may be an effective method of protecting SB for STEMI patients involving coronary artery bifurcation and underwent emergent PCI.

## Introduction

Coronary artery bifurcation disease (CABD) occurs in 15%–20% of patients with coronary artery disease (CAD) undergoing percutaneous coronary intervention (PCI) (1, 2), and it also occurs in 10%–20% of cases with ST-elevation myocardial infarction (STEMI) under primary PCI (3, 4). Treatment of bifurcation lesions has a lower procedural success rate and higher long-term adverse outcomes when compared with non-bifurcation lesions. The best approach for treating bifurcation lesions remains controversial. Contemporary practice has favors the provisional one-stent strategy over the two-stent strategy. Whereas the best way to preserve a side branch (SB) remains unresolved. Some current methods are applied in SB protection when a provisional one-stent strategy is performed in CABD (5–10). A protection guidewire or a jailed balloon placed inside the SB prior to the main vessel (MV) stenting provides SB protection for CABD PCI. The jailed semi-inflated balloon technique (JSIBT) was first introduced in 2015 by Cayli et al. and was shown to reduce acute SB occlusion at the time of implanting a MV drug-eluting stent (DES) or the bioresorbable scaffolds (BRS) (5–9, 11). However, these methods of SB protection are typically applied to CABD PCI limited to the setting of stable/chronic CAD, but not to the acute setting of STEMI. To date, no study has addressed the use of JSIBT in STEMI patients treated with emergent PCI for a culprit lesion involving a coronary artery bifurcation. Here, we aimed to investigate, based on acute angiography, the efficacy and safety of JSIBT for SB protection in STEMI patients who underwent emergent PCI for a culprit lesion involving a coronary artery bifurcation.

## Materials and methods

### Study population

We retrospectively enrolled and analyzed, between Jan, 2011 and Jun, 2020, STEMI patients with a culprit lesion involving a coronary artery bifurcation undergoing emergent PCI with SB protection by JSIBT or a wire, or without SB protection. We excluded those patients with initial presentation of cardiogenic shock/arrest on admission, a past history of CABG, stent thrombosis at a coronary artery bifurcation. Written informed consent for emergent PCI was obtained from each patient. Their baseline demographic data were retrospectively reviewed through medical records of the hospital, and interventional procedures and immediate angiographic findings after PCI were carefully reviewed by two experienced interventional cardiologists. The study protocol was approved by the Institutional Review Board/Ethics Committee of Taichung Veterans General Hospital, Taichung, Taiwan (TCVGH IRB NO. CE21249B).

### Intervention procedures

**Figure 1:**
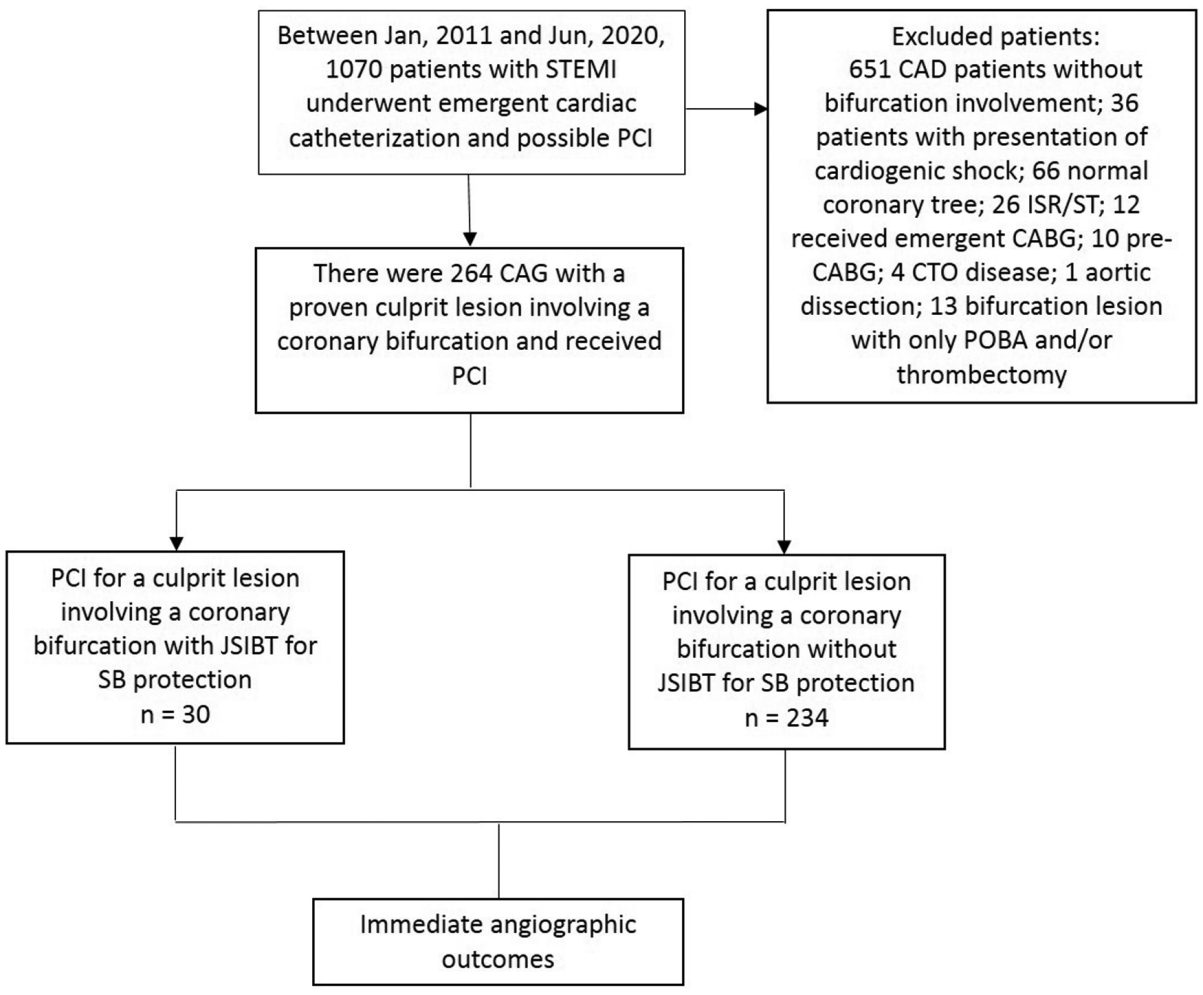
Flow chart of STEMI patients with a culprit lesion involving a coronary artery bifurcation who underwent primary PCI in our institute.

All procedures were carried out using the standard PCI protocols of our catheter laboratory. The patients each received a loading dose of aspirin (300 mg), and either clopidogrel (600 mg), ticagrelor (180 mg) or prasugrel (20 mg), at the operator’s discretion prior to or at the time of emergent PCI. All patients received heparin anticoagulants including a loading dose and continuous dose during the procedure, with a targeted ACT of 300”. The use of glycoprotein IIb/IIIa inhibitors was at the operator’s discretion. The procedure of JSIBT for CABD was described in detail previously (9, 11). Figure 1 illustrates steps of JSIBT for our patients in a setting of acute STEMI. A culprit lesion was either wired and dilated with a small size balloon, or aspirated with a manual aspiration catheter. After the TIMI flow had been restored or improved, a second wire was placed into the SB. The stent was subsequently advanced into the MV, and positioned over the bifurcation lesion. Thereafter, a smaller semi-compliant quarter-size balloon, or one of an equal size, was advanced into the SB beforehand, ensuring 1–2 mm protrusion of the proximal balloon into the MV. The protection balloon in the SB was inflated at low pressure (typically between 4 and 6 atm). Finally the MV stent was deployed slowly at a sub-nominal pressure, jailing the semi-inflated SB balloon. During implantation of the MV stent, the SB balloon was kept inflated. Both the SB balloon and the MV stent balloon were then simultaneously deflated and the SB balloon was removed. The MV stent balloon was reinflated at nominal pressure to restore the deformed stent and to fully expand it. In the final step, both a post-dilatation of the whole stent with a non-compliant balloon and a proximal optimal dilatation therapy (POT) of the stent segment were performed to achieve good stent apposition to the MV arterial wall. No rewiring of the SB was done if angiography showed SB patency. However, in the event of acute occlusion or imminent jailing, SB was rewired and the kissing balloon technique (KBT) was used to restore SB flow.

### Definition of study endpoints

The primary study endpoints in this study were SB complications, as well as the MV and SB TIMI flows measured during the peri-procedure and post-procedure periods. SB complications included SB dissection, SB occlusion, balloon rupture/entrapment, or wire entrapment. MV and SB TIMI flows of the post-procedure period were evaluated when the emergent PCI had been completed. All endpoints were chosen to assess the efficacy and safety of JSIBT for SB protection.

### Statistical analysis

Continuous variables were presented as median with interquartile range due to their non-normal distribution. Categorical variables were presented as number and percentage. Continuous variables of the two groups were compared with the Mann-Whitney *U* test. Categorical variables were compared with Chi-square test or Fisher exact test. Pre- and post-procedure quantitative coronary angiography (QCA) TIMI flows were compared using the Wilcoxon signed rank test for the individual group. Statistical significance was set at *p* < 0.05. All statistical analyses were performed using SPSS software 19.0 (SPSS Inc., Chicago, Illinois, USA).

## Results

### Baseline characteristics of STEMI patients with primary PCI for a culprit lesion involving a coronary artery bifurcation

**Figure 2:**
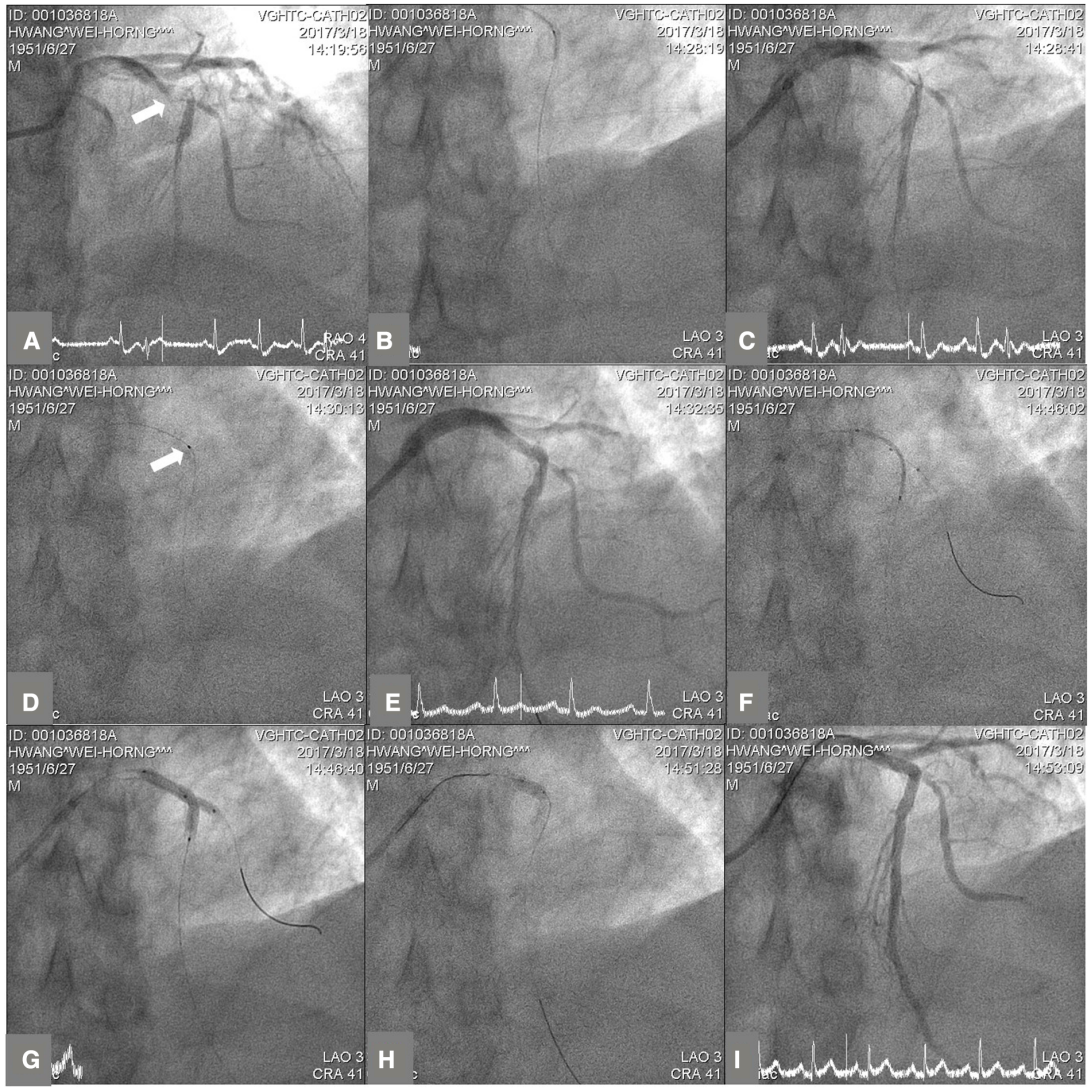
The steps of side branch protection with jailed semi-inflated balloon technique (JSIBT) for a culprit lesion involving a coronary artery bifurcation in a STEMI patient. (**A**) Diagnostic coronary angiography (CAG) at antero-posterior and cranial 41° projection showed a culprit lesion at proximal left anterior descending (LAD) artery bifurcation (white arrow). (**B**) Wiring of the main vessel (MV) LAD and balloon dilatation of the MV. (**C**) CAG post-balloon dilatation of the MV revealed a soft plaque and thrombus at proximal LAD with extension to the orifice of diagonal side branch (SB). (**D**) Thrombus suction by an aspiration catheter (white arrow). (**E**) CAG post-aspiration thrombectomy of the MV revealed haziness at the LAD proximal-middle bifurcation and orifice of the SB. (**F**) Placement of a semi-compliant balloon in the diagonal SB and a DES in proximal to middle LAD, covering the MV lesion. (**G**) DES and a semi-compliant balloon were inflated simultaneously. The SB balloon was inflated at low pressure (6 atmospheres) and the DES less than nominal pressures. (**H**) For optimization of the MV stent, proximal optimal technique was performed with a short non-compliant balloon. (**I**) Final CAG at LAO 3° and cranial 41° projection showed a good angiographic result and good flows of the MV and SB.

Between January 2011 and June 2020, there were 1070 patients with STEMI undergoing emergent cardiac catheterization (CAG) and possible PCI. Among them, 264 patients received CAG, which disclosed a culprit lesion involving a coronary artery bifurcation. These patients then received PCI, and were enrolled in our study. Figure 2 shows the flow chart. Their baseline patient characteristics are shown in Table 1. Among them, 30 STEMI patients with a culprit lesion involving a coronary artery bifurcation were treated with JSBIT for SB protection during primary PCI, and the remaining 234 patients either used or not used a wire for SB protection. Patients in the JSIBT group had a higher prevalence of DM and concomitant left main disease compared with the non-JSIBT group. Similar between the two groups were their distributions of gender, age, background underlying diseases, and risk factor profiles (except for DM), clinical presentations and diagnosis, and characteristics of the culprit at a coronary artery bifurcation.

**Table 1:** Demographic characteristics of STEMI patients with a culprit lesion involved a coronary bifurcation receiving primary PCI utilizing jailed semi-inflated balloon technique for side branch protection or not.

	JSIBT group	Non-JSIBT group	*P* value
	*n* = 30	*n* = 234	
Gender M/F (*N*, %)	28/2 (93.3/6.7)	199/35 (85/15)	0.219
Age (years)	61 (58, 77)	63 (52, 72)	0.296
Hypertension (*N*, %)	22 (73.3)	143 (61.1)	0.194
Diabetes mellitus (*N*, %)	16 (53.3)	71 (30.3)	0.012
Statin for dyslipidemia (*N*, %)	22 (73.3)	160 (68.4)	0.581
Smoking (*N*, %)	22 (73.3)	138 (59.0)	0.130
Prior PCI (*N*, %)	0 (0)	20 (8.5)	0.096
Prior MI (*N*, %)	0 (0)	15 (6.4)	0.154
Old CVA (*N*, %)	6 (20.0)	23 (9.8)	0.094
Admission diagnosis (*N*, %)			0.534
STEMI	30 (100)	231 (99)	
NSTEMI	0 (0)	3 (1)	
CKD (*N*, %)	4 (13.3)	31 (13.2)	0.990
BMI (kg/m^2^)	25.3 (22.4, 28.5)	24.6 (22.9, 27.24)	0.591
Hemoglobin (mg/dl)	15.4 (13.8, 16.8)	14.9 (13.4, 16.2)	0.547
Total cholesterol (mg/dl)	170.0 (145.0, 215.0)	171.0 (152.0, 200.5)	0.842
Triglyceride (mg/dl)	108.0 (66.0, 161.0)	105.0 (62.3, 178.8)	0.722
HDL-C (mg/dl)	40.0 (39.0, 47.0)	42.0 (36.0, 49.5)	0.942
LDL-C (mg/dl)	99.0 (87.0, 150.0)	109.5 (90.3, 134.8)	0.998
BUN (mg/dl)	19.0 (12.0, 24.0)	18.0 (14.0, 24.8)	0.747
Creatinine (mg/dl)	0.95 (0.82, 1.29)	1.00 (0.83, 1.24)	0.439
LVEF (%)	42.5 (37.0, 50.0)	41.0 (36.0, 47.3)	0.601
Severity of CAD			
Vessel numbers (*N*)	1.0 (1, 2)	1.0 (1, 2.0)	0.980
MVD (*N*, %)	12 (40.0)	98 (41.9)	0.844
Left main disease (*N*, %)	4 (13.3)	7 (3.0)	0.008
Bifurcation lesion Location			0.056
LM (*N*, %)	0 (0)	4 (1.7)	
LAD (*N*, %)	28 (93.3)	177 (75.6)	
LCX (*N*, %)	2 (6.7)	33 (14.1)	
RCA (*N*, %)	0 (0)	20 (8.5)	

Data are presented as median (interquartile range) for continuous variables and *n* (%) for categorical variables. Chi-square or Fisher exact test for categorical variables and a Mann-Whitney *U* test for continuous variables.

STEMI, ST segment elevation myocardial infarction; PCI, percutaneous coronary intervention; JSIBT, jailed semi-inflated balloon technique; MI, myocardial infarction; CVA, cerebrovascular accident; NSTEMI, non-ST segment elevation myocardial infarction; CKD, chronic kidney disease; BMI, body mass index; HDL-C, high-density lipoprotein cholesterol; LDL-C, low-density lipoprotein cholesterol; BUN, blood urea nitrogen; LVEF, left ventricular ejection fraction; CAD, coronary artery disease; MVD, multiple vessel disease; LM, left main; LAD, left anterior descending artery; LCX, left circumflex artery; RCA, right coronary artery.

### Interventional and coronary angiographic characteristics of JSIBT for primary PCI for a culprit lesion involving a coronary artery bifurcation

Interventional and quantitative coronary angiographic characteristics are shown in Table 2, and QCA results for MV and SB at baseline and post-procedure are shown in Table 3. The culprit lesions were similar between the two groups in length and size. Also similar were the two groups’ SB vessel size, MV stent length, size and type, as well as SB balloon size. In the JSIBT, more patients received PCI using 6 Fr guide catheter via a transradial approach, and their SB balloon was shorter in length (12.0 vs. 20.0 mm, *p* < 0.001) and used a lower inflation pressure (6 vs. 8 atm, *p* < 0.001) compared with those used in the non-JSIBT group. The use of a wire for SB protection was higher in the JSIBT group. POT was performed 100% in the JSIBT group, a percentage which was significantly higher than that in the non-JSIBT group (100% vs. 39.3%, *p* < 0.001). Moreover, their balloon size for POT was significantly bigger than that in the non-JSIBT group (3.25 vs. 3.0 mm, *p* = 0.003). In the non-JSIBT incidence rates of SB rewiring (6.7% vs. 26.9%, *p* = 0.013) and kissing balloon technique (6.7% vs. 25.2%, *p* = 0.021) were significantly higher compared with the JSIBT group. In both groups, post-interventional MV and peri-/post-interventional SB TIMI flow values were significantly higher compared with their pre-interventional values. Peri-interventional (93.3% vs. 60.7%, *p* < 0.001) and post-interventional SB TIMI 3 flows (96.7% vs. 77.8%, *p* = 0.041) were significantly higher in the JSIBT group compared with the non-JSIBT group. Post-interventional MV TIMI flow values were similar between the two groups (3 vs. 3, *p* = 0.129). In both groups, no SB balloon rupture or entrapment nor wire entrapment had occurred. Patients in the JSIBT group had a significantly greater prevalence of SB dissection (6 of 30; 20%) compared with the non-JSIBT group (19 of 234; 8.1%; *p* = 0.048), but no significantly lower prevalence of SB occlusion compared with the non-JSIBT group (0 vs. 19 patients, *p* = 0.145). Ten patients in the non-JSIBT group required a 2-stent strategy, and the inter-group difference was not statistically significant, despite no patient having such a requirement in the JSIBT group.

**Table 2:** Interventional and quantitative coronary angiographic characteristics of STEMI patients with a culprit lesion involving a coronary bifurcation receiving primary PCI with or without jailed semi-inflated balloon technique for side branch protection.

	JSIBT group	Non-JSIBT group	*P* value
	*n* = 30	*n* = 234	
Guide size			0.035
6 (*N*, %)	28 (93.3)	176 (75.2)	
7 (*N*, %)	2 (6.7)	58 (24.8)	
Approach (Radial/Femoral) (*N*, %)	28/2 (93.3/6.7)	163/71 (69.7/30.3)	0.004
Thrombectomy in PCI (*N*, %)	22 (73.3)	139 (59.4)	0.167
IVUS or OCT in PCI (*N*, %)	4 (13.3)	14 (6.0)	0.133
SB wire protection	30 (100)	127 (54.3)	<0.001
SB balloon pre-dilatation	10 (33.3)	85 (36.3)	0.841
Stent type			0.232
BMS (*N*, %)	8 (26.7)	91 (38.9)	
DES (*N*, %)	22 (73.3)	143 (61.1)	
Culprit lesion			
Length (mm)	22.9 (15.9, 40.5)	24.2 (18.9, 32.4)	0.953
Size (mm)	3.13 (2.80, 3.80)	3.11 (2.87, 3.52)	0.686
SB vessel size (mm)	2.21 (2.00, 2.30)	2.17 (1.94, 2.40)	0.561
Main vessel stent			
Size (mm)	3.00 (2.50, 3.50)	2.75 (2.75, 3.00)	0.912
Length (mm)	26.0 (18.0, 38.0)	28.0 (23.0, 38.0)	0.242
SB balloon (*N*, %))	30 (100)	60 (25.6)	
Size (mm)	2.00 (2.00, 2.50)	2.00 (2.00, 2.50)	0.246
Length (mm)	12.0 (12.0, 12.0)	20.0 (20.0, 20.0)	<0.001
Inflation pressure (atm)	6 (6, 8)	8 (8, 10)	<0.001
Proximal optimal dilatation (*N*, %)	30 (100)	92 (39.3)	<0.001
BC size (mm)	3.25 (3.0, 3.75)	3.0 (2.75, 3.50)	0.003
SB rewiring (*N*, %)	2 (6.7)	63 (26.9)	0.013
Kissing balloon technique (*N*, %)	2 (6.7)	59 (25.2)	0.021

Data are presented as median (interquartile range) for continuous variables and *n* (%) for categorical variables. Chi-square or Fisher exact test for categorical variables and a Mann-Whitney *U* test for continuous variables.

STEMI, ST segment elevation myocardial infarction; PCI, percutaneous coronary intervention; JSIBT, jailed semi-inflated balloon technique; IVUS, intravascular ultrasound; OCT, optical coherence tomography; SB, side branch; BMS, bare-metal stent; DES, drug-eluting stent; BC, balloon catheter.

**Table 3:** Angiographic results of a culprit lesion involving a coronary bifurcation in STEMI patients before and after receiving primary PCI with or without jailed semi-inflated balloon technique for side branch protection.

	JSIBT group	Non-JSIBT group	*P* value
	*n* = 30	*n* = 234	
**Pre-interventional TIMI flow**			
MV TIMI flow			
Median (Q25, Q75)	2 (1, 2)	1 (0, 2)	0.129
TIMI 0 (*N*, %)	4 (13.3)	106 (45.3)	
TIMI 1 (*N*, %)	10 (33.3)	20 (8.5)	
TIMI 2 (*N*, %)	12 (40.0)	59 (25.2)	
TIMI 3 (*N*, %)	4 (13.3)	49 (20.9)	
SB TIMI flow			
Median (Q25, Q75)	2 (1, 3)	2 (0, 3)	0.432
TIMI 0 (*N*, %)	6 (20.0)	81 (34.6)	
TIMI 1 (*N*, %)	2 (6.7)	17 (7.3)	
TIMI 2 (*N*, %)	10 (33.3)	37 (15.8)	
TIMI 3 (*N*, %)	12 (40.0)	99 (42.3)	
**Peri-interventional SB TIMI flow**			
Median (Q25, Q75)	3 (3, 3)*	3 (2, 3)^‡^	<0.001
TIMI 0 (*N*, %)	0 (0)	12 (5.1)	
TIMI 1 (*N*, %)	0 (0)	19 (8.1)	
TIMI 2 (*N*, %)	2 (6.7)	61 (26.1)1	
TIMI 3 (*N*, %)	28 (93.3)	142 (60.7)	
**Post-interventional TIMI flow**			
MV TIMI flow			
Median (Q25, Q75)	3 (3, 3)^†^	3 (3, 3)^§^	0.129
TIMI 0 (*N*, %)	0 (0)	1 (4)	
TIMI 1 (*N*, %)	0 (0)	0 (0)	
TIMI 2 (*N*, %)	1 (3.3)	29 (12.4)	
TIMI 3 (*N*, %)	29 (96.7)	204 (87.2)	
SB TIMI flow			
Median (Q25, Q75)	3 (3,3)^+^	3 (3,3)^£^	0.041
TIMI 0 (*N*, %)	0 (0) 12	(5.1)	
TIMI 1 (*N*, %)	0 (0)	9 (3.8)	
TIMI 2 (*N*, %)	1 (3.3)	31 (13.2)	
TIMI3 (*N*, %)	29 (96.7)	182 (77.8)	
**SB complications**	6 (20.0)	37 (15.8)	0.599
Dissection (*N*, %)	6 (20.0)	19 (8.1)	0.048
Occlusion (*N*, %)	0 (0)	21 (9.0)	0.145
BC rupture/entrapment	0 (0)	0 (0)	−
Wire entrapment	0 (0)	0 (0)	−
**2-stent strategy needed**	0 (0)	10 (4.3)	0.610
Stent length, mm (Q25, Q75)	−	21.5 (17.3, 28.0)	
Stent size, mm (Q25, Q75)	−	2.5 (2.4, 3.0)	

Data are presented as median (interquartile range) for continuous variables and *n* (%) for categorical variables. Chi-square or Fisher exact test for categorical variables and a Mann-Whitney U test for continuous variables.

STEMI, ST segment elevation myocardial infarction; PCI, percutaneous coronary intervention; JSIBT, jailed semi-inflated balloon technique; TIMI, thrombolysis in myocardial infarction; MV, main vessel; SB, side branch; BC, balloon catheter.

^*,†,‡,§,+,£^ Analysis of TIMI flow pre-, peri- and post-procedure were compared using the Wilcoxon signed rank test in each group; *, †, ‡, §, +, £ also indicated *P* value < 0.001.

### Predictors of SB complications in STEMI patients with a culprit lesion involving a coronary artery bifurcation underwent primary PCI

Results of univariate and multivariate logistic regression analyses are presented in Table 4, showing possible predictors of SB complications in our STEMI patients who underwent PCI for a culprit lesion involving a coronary artery bifurcation. Background factors (age and male gender), angiographic and interventional factors (SB wire protection and balloon predilattion) were not independent predictors for SB complications, while the use of DES was a borderline independent predictor of SB complications (Odds ratio 0.364, 95% confidence interval 0.131–1.006, *p* = 0.051).

**Table 4:** Logistic regression analysis for side branch complications in all STEMI patients with a culprit lesion involving a coronary bifurcation receiving primary PCI.

	Univariate			Multivariate		
Odds ratio	*P* value	95% CI	Odds ratio	*P* value	95% CI
Age	1.036	0.010	1.008–1.064	1.021	0.284	0.983–1.062
Male gender	0.388	0.020	0.175–0.861	0.793	0.774	0.163–3.862
Bifurcation location	1.889	0.628	0.144–24.793	—	—	—
JSIBT	1.331	0.560	0.509–3.480	—	—	—
DES	0.510	0.045	0.264–0.986	0.364	0.051	0.131–1.006
Thrombectomy in PCI	1.096	0.791	0.538–2.151	—	—	—
POT	1.268	0.477	0.659–2.438	—	—	—
POT balloon size	3.111	0.073	0.898–10.772	2.132	0.274	0.549–8.277
SB wire protection	2.234	0.032	1.071–4.659	1.223	0.787	0.285–5.238
SB balloon predilatation	2.124	0.025	1.097–4.111	2.377	0.152	0.727–7.767
Hypertension	1.677	0.159	0.817–3.442	—	—	—
Diabetes mellitus	1.252	0.517	0.634–2.471	—	—	—
Hyperlipidemia with statin therapy	1.377	0.397	0.656–2.889	—	—	—
Smoking	0.705	0.298	0.365–1.361	—	—	—
MV stent size	0.493	0.148	0.190–1.284	—	—	—
MV size	0.972	0.934	0.490–1.928	—	—	—
SB vessel size	0.626	0.341	0.239–1.640	—	—	—
SB balloon size	0.438	0.323	0.085–2.251	—	—	—
SB balloon inflation pressure	1.092	0.500	0.846–1.410	—	—	—
SB TIMI flow pre-procedure	0.885	0.332	0.691–1.133	—	—	—

STEMI, ST segment elevation myocardial infarction; PCI, percutaneous coronary intervention; JSIBT, jailed semi-inflated balloon technique; DES, Drug-eluting stent; POT, proximal optimal technique; SB, side branch; MV, main vessel; TIMI, thrombolysis in myocardial infarction.

### Predictors of MV and SB TIMI 3 flows in the post-procedure period for STEMI patients with a culprit lesion involving a coronary artery bifurcation who underwent primary PCI

Results of univariate and multivariate logistic regression analyses are presented in Tables 5, 6, showing possible predictors of MV and SB TIMI 3 flows post-procedure after PCI in all STEMI patients with a culprit lesion involving a coronary artery bifurcation. MV lesion length (Odds ratio 0.966, 95% confidence interval 0.938–0.994, *p* = 0.019) and MV TIMI flow pre-procedure (Odds ratio 1.604, 95% confidence interval 1.120–2.298, *p* = 0.010) were independent predictors for MV TIMI 3 flow values at the post-procedure period after primary PCI, and male gender (Odds ratio 3.165, 95% confidence interval 1.448–6.919, *p* = 0.004), JSIBT (Odds ratio 11.363, 95% confidence interval 1.352–95.543, *p* = 0.025), and KBT (Odds ratio 4.405, 95% confidence interval 1.472–13.184, *p* = 0.008) were independent predictors for SB TIMI 3 flow values at the post-procedure period by the end of primary PCI.

**Table 5:** Logistic regression analysis for final main vessel TIMI 3 flow in all STEMI patients with a culprit lesion involving a coronary bifurcation receiving primary PCI.

	Univariate			Multivariate		
	Odds ratio	*P* value	95% CI	Odds ratio	*P* value	95% CI
Age	1.012	0.434	0.982–1.042	—	—	—
Male gender	1.564	0.365	0.594–4.117	—	—	—
Bifurcation location	3.000	0.424	0.203–44.359	—	—	—
JSIBT	4.265	0.161	0.560–32.471	—	—	—
DES	1.234	0.588	0.577–2.643	—	—	—
Thrombectomy in PCI	1.332	0.456	0.626–2.835	—	—	—
POT	0.906	0.796	0.428–1.918	—	—	—
POT balloon size	1.265	0.687	0.403–3.972	—	—	—
SB wire protection	1.240	0.577	0.583–2.636	—	—	—
SB balloon predilatation	0.752	0.464	0.351–1.612	—	—	—
Hypertension	0.770	0.522	0.347–1.711	—	—	—
Diabetes mellitus	0.644	0.260	0.300–1.385	—	—	—
Hyperlipidemia with statin therapy	1.065	0.878	0.477–2.376	—	—	—
Smoking	1.517	0.278	0.715–3.219	—	—	—
MV size	1.809	0.176	0.766–4.272	—	—	—
MV stent size	2.498	0.115	0.797–7.831	—	—	—
MV stent length	0.984	0.255	0.958–1.011	—	—	—
MV lesion length	0.969	0.058	0.943–1.023	0.966	0.019	0.938–0.994
KBT	1.034	0.941	0.423–2.532	—	—	—
MV TIMI flow pre-procedure	1.566	0.014	1.097–2.236	1.604	0.010	1.120–2.298

TIMI, thrombolysis in myocardial infarction; STEMI, ST segment elevation myocardial infarction; PCI, percutaneous coronary intervention; JSIBT, jailed semi-inflated balloon technique; DES, drug-eluting stent; POT, proximal optimal technique; SB, side branch; MV, main vessel; KBT, kissing balloon technique.

**Table 6:** Logistic regression analysis for final side branch TIMI 3 flow in all STEMI patients with a culprit lesion involving a coronary bifurcation receiving primary PCI.

	Univariate			Multivariate		
	Odds ratio	*P* value	95%	CI Odds ratio	*P* value	95% CI
Age	1.012	0.335	0.988–1.036	—	—	—
Male gender	3.391	0.001	1.613–7.129	3.165	0.004	1.448–6.919
Bifurcation location	0.619	0.700	0.054–7.121	—	—	—
JSIBT	8.286	0.040	1.102–62.283	11.363	0.025	1.352–95.543
DES	1.653	0.106	0.899–3.039	—	—	—
Thrombectomy in PCI	0.762	0.400	0.405–1.434	—	—	—
POT	1.890	0.047	1.007–3.546	0.785	0.519	0.376–1.639
POT balloon size	0.970	0.956	0.322–2.919	—	—	—
SB wire protection	1.700	0.086	0.928–3.117	0.939	0.854	0.482–1.831
SB balloon predilatation	1.117	0.732	0.593–2.106	—	—	—
Hypertension	1.235	0.500	0.668–2.282	—	—	—
Diabetes mellitus	1.662	0.147	0.836–3.302	—	—	—
Hyperlipidemia with statin therapy	0.950	0.878	0.494–1.828	—	—	—
Smoking	1.230	0.505	0.669–2.264	—	—	—
MV stent size	0.774	0.515	0.358–1.673	—	—	—
MV vessel size	0.647	0.162	0.351–1.191	—	—	—
SB vessel size	1.412	0.439	0.589–3.387	—	—	—
SB vessel balloon size	0.839	0.885	0.079–8.915	—	—	—
SB balloon inflation pressure	0.837	0.386	0.561–1.250	—	—	—
KBT	3.468	0.012	1.314–9.153	4.405	0.008	1.472–13.184
SB TIMI flow pre-procedure	1.202	0.114	0.957–1.511	—	—	—

TIMI, thrombolysis in myocardial infarction; STEMI, ST segment elevation myocardial infarction; PCI, percutaneous coronary intervention; JSIBT, jailed semi-inflated balloon technique; DES, drug-eluting stent; POT, proximal optimal technique; SB, side branch; MV, main vessel; KBT, kissing balloon technique.

## Discussion

Our principal finding is the use of JSIBT in our patients (STEMI with a culprit lesion involving a coronary artery bifurcation undergoing emergent PCI) had provided an effective SB protection, good acute procedural outcomes, and a better final SB TIMI 3 flow, as compared to the strategy of SB protection using or not using a placed guide wire. Despite the higher prevalence of SB dissection in the JSIBT group, total SB complications were similar to those with SB protection by using or not using a placed guide wire. Results indicated that JSIBT is likely safe for this cohort.

CABD occurs in 15%–20% and 10%–20% of CAD patients undergoing elective and primary PCIs (1–4) and has consistently higher rates of procedural complications and adverse cardiovascular outcomes when compared with similar non-CABD cases, even in the DES era (8, 12–15). PCI of CABD is associated with greater prevalence of SB occlusion, peri-procedural MI, and poorer clinical outcomes. These consequences are explained in terms of target lesion re-vascularization and stent thrombosis, and the complex anatomy and dynamic nature of bifurcation lesions. Such lesions are prone, during PCI, to form plaques, or induce carina shift, changes in bifurcation angles, vessel spasm, and/or SB dissection/occlusion. Jailed wire technique, jailed balloon technique (JBT), and JSIBT have been applied to protect SB during PCI of CABD. They have led to development of various standards or other novel SB protection techniques (5–10). Our present study determined the efficacy of JSIBT for SB protection of STEMI patients with a culprit lesion involving a coronary artery bifurcation. Our results showed that JSIBT had achieved a higher SB TIMI flow during both peri- and post-procedure periods, as compared with pre-procedural measurements. In addition, JSIBT had better SB TIMI flows during and post PCI as compared with the non-JSIBT group. JSIBT also predicted well post-procedural SB TIMI 3 flow values. Regarding safety, despite the higher prevalence of SB dissection with JSBIT, we found no difference in SB complications in terms of SB dissection, SB occlusion, BC rupture/entrapment or wire entrapment. The percentages of wire placement in the SB branch, with simultaneous deployment of a MV stent and SB branch balloon inflation, were all 100% in the JSIBT group, but were 54.3% and 25.2% in the non-JSIBT group. There findings likely can explain for the higher rate of SB dissection in the JSIBT group. Even though there was a higher SB dissection rate in the JSIBT group, the prevalence rates of SB occlusion and total SB complications were 0% and 20% respectively in the JSIBT group, compared with 9% and 15.8% in the non-JSIBT group. We found no inter-group difference on these values. In contrast to the traditional application of SB protection using a placed wire or bail-out rewiring with KBT in complex CABD PCI, JSIBT not only provided a better SB protection with final SB TIMI 3 flow during primary PCI, but it also had a similar rate of SB complications compared to those with or without the use of SB wire protection. Results are in support of the safety of JSIBT in clinical practice.

JSIBT was as an extension of the jailed wire technique and JBT, and was first introduced in 2015 by Cayli et al., (9, 16) who performed provisional one-stent strategy with JSIBT for CABD on 148 lesions in 137 patients. Among these patients, 64.2% had ACS and 73.7% had true bifurcation lesions. TIMI 3 blood flow after MV stent placement was 100% with both MV and SB, with no SB occlusion, and no rupture or entrapment of inflated-balloon. We revealed similar immediate angiographic findings with 96.7% of TIMI 3 flow from both MV and SB as measured post-procedure, with no SB occlusion, and no rupture nor entrapment of inflated-balloon in all our STEMI patients. However, our prevalence of SB dissection was 20%, which was higher compared with the 2.7% as reported by Cayli et al. in a similar study. The discrepancy in results may be due to our higher inflation pressure of jailed balloon in the SB. Since the pathophysiological nature of plaque in the setting of STEMI is soft and thrombus-like, and the SB is essentially non-diseased, we recommended that the inflation pressure of the SB balloon could be set lower than the officially recommended pressure in order to reduce risks of SB dissection.

Our study had some limitations. Firstly, this was a non-randomized, retrospective, observational and case cohort study. It was therefore subject to all the limitations inherent in the study design. Secondly, the study populations in both groups were relatively small and patient selections in the two groups were heterogeneous. In the non-JSIBT group, 54.3% used jailed wire technique for SB protection, 26.9% required SB rewiring, and 25.2% completed PCI with KBT. In contrast, all patients in the JSIBT group received wire placement into the SB with inflation of a semi-inflated balloon at the time of MV stent deployment to complete the PCI. However, our study was focused on the efficacy and safety of a recently applied approach for SB protection using JSIBT in STEMI patients with a culprit lesion involving a coronary artery bifurcation. Currently, there is no guideline for this group of patients and no clue to predict which lesion type requires the placement of a wire in the SB. The choice was entirely at the discretion of operators, typically based on their individual experiences. The heterogeneity of our studied STEMI patients in the two groups might truly reflect real-world practice. Our results demonstrated that JSIBT is useful for SB protection with an acceptable level of SB complications in STEMI patients with a culprit lesion involving a coronary artery bifurcation and receiving primary PCI. Further studies on a larger population and using a randomized control design, are needed to confirm our present conclusion.

## Conclusion

JSIBT used as a method of SB protection during primary PCI not only provided better SB protection, but it also had a similar rate of SB complications in comparison with prior use of SB wire protection. Thus, JSIBT is likely a novel technique for effectively protecting SB during primary PCI for a culprit lesion involving a coronary artery bifurcation in STEMI patients. Specially, this novel technique provided a remarkable final SB TIMI 3 flow with non-complicated SB dissection, particularly using a lower inflation pressure of the SB balloon.

## Data Availability

The original contributions presented in the study are included in the article, further inquiries can be directed to the corresponding author.
